# Waiting time for surgery influences the outcome in idiopathic normal pressure hydrocephalus — a population-based study

**DOI:** 10.1007/s00701-021-05085-7

**Published:** 2021-12-30

**Authors:** Christine Chidiac, N. Sundström, M. Tullberg, L. Arvidsson, M. Olivecrona

**Affiliations:** 1 Department of Medical Sciences, Faculty of Medicine and Health, Örebro University, Örebro, Sweden; 2 Department of Radiation Sciences, Radiation Physics, Biomedical Engineering, Umeå University, Umeå, Sweden; 3 Hydrocephalus Research Unit, Department of Clinical Neuroscience, Institute of Neuroscience and Physiology, Sahlgrenska Academy, Sahlgrenska University Hospital, University of Gothenburg, Gothenburg, Sweden; 4 Department of Clinical Neuroscience Karolinska Institutet, Department of Neurosurgery, Karolinska University Hospital, Stockholm, Sweden; 5 Department of Neurosurgery, Faculty of Medicine and Health, Örebro University, Örebro, Sweden

**Keywords:** Idiopathic normal pressure hydrocephalus, Waiting time, Shunt surgery, iNPH scale, Sex

## Abstract

**Introduction:**

Idiopathic normal pressure hydrocephalus (iNPH) is a disease that comes with a great impact on the patient’s life. The only treatment for iNPH, which is a progressive disease, is shunt surgery. It is previously indicated that early intervention might be of importance for the outcome.

**Aim:**

To investigate if a longer waiting time for surgery, negatively influences the clinical outcome.

**Methods:**

Eligible for this study were all iNPH patients (n = 3007) registered in the Swedish Hydrocephalus Quality Registry (SHQR) during 1^st^ of January 2004–12^th^ of June 2019. Waiting time, defined as time between the decision to accept a patient for surgery and shunt surgery, was divided into the intervals ≤ 3, 3.1–5.9 and ≥ 6 months. Clinical outcome was assessed 3 and 12 months after surgery using the modified iNPH scale, the Timed Up and Go (TUG) test and the mini mental state examination (MMSE).

**Results:**

Three months after surgery, 57% of the patients with ≤ 3 months waiting time showed an improvement in modified iNPH scale (≥ 5 points) whereas 52% and 46% of patients with 3.1–5.9 and ≥ 6 months waiting time respectively improved (p = 0.0115). At 12 months of follow-up, the corresponding numbers were 61%, 52% and 51% respectively (p = 0.0536).

**Conclusions:**

This population-based study showed that in patients with iNPH, shunt surgery should be performed within 3 months of decision to surgery, to attain the best outcome.

## Introduction

First described by Hakim and Adams in 1965, idiopathic normal pressure hydrocephalus (iNPH) is the most common form of hydrocephalus in adults, afflicting 1–4% of those over 65 years of age [1, 6, 8, 9, 17]. The brain disorder iNPH with its insidious onset and gradual progression has an average onset at 70 years of age [20]. Symptoms, sometimes denoted Hakim’s triad, comprise gait and balance impairment, dementia, and urinary incontinence [6, 14]; however, a formal definition does not exist. The disease is effectively treated with a cerebrospinal fluid shunt [2, 10].

The Swedish Hydrocephalus Quality Registry (SHQR) started in 2004 and contains standardized data on clinical features, surgical procedures, and follow-up (at 3 and 12 months) of all patients, 18 years or older, operated for hydrocephalus in Sweden [16].

At present, six of the seven neurosurgical units in Sweden (Gothenburg, Linkoping, Orebro, Stockholm, Umea, and Uppsala) prospectively include operated patients with a close to 100% coverage. Stockholm joined in 2013 and stands for a part of the non-included patients together with Lund which in 2017 dropped out from the register. Total coverage of the operated iNPH patients in Sweden is estimated to be 80% [16].

Since iNPH is a progressive disease where clinical symptoms are known to worsen over time, it is reasonable to assume that early treatment has the potential to preserve functionality and lead to a better postoperative outcome. The progressive nature of the disease would mean that waiting time for shunt surgery should be kept at a minimum. A previous study comparing 69 patients who waited less than 3 months (median 0.2 month) for shunt surgery with 33 patients who waited longer than 6 months (median 13.2 months) showed that early surgery provides the greatest potential for postoperative improvement, concluding that intervention should be performed soon after diagnosis [2]. The influence of waiting time for shunt surgery on outcome has not been reported in larger studies.

### Aim

The aim was on a population-basis using data from SHQR, to investigate if a longer time between the decision to accept the patient for shunt surgery and the surgical procedure, i.e. prolonged waiting time for surgery, negatively influences the clinical outcome in patients with iNPH.

## Material and methods

### Patients

All patients diagnosed with iNPH according to international guidelines [14], operated and registered in the SHQR during 1^th^ of January 2004–12^th^ of June 2019, when data for this study were extracted, were included. Waiting time, defined as the time between decision to accept a patient for surgery and the surgical procedure, was divided into the intervals short (≤ 3 months), intermediate (3.1–5.9) and long (≥ 6) months. Waiting times 3 years or longer were considered to be outliers and were excluded from analyses.

### The iNPH scale

The iNPH scale was introduced by Hellström et al. in order to evaluate severity and outcome in iNPH patients [7]. It covers the four most important symtoms; gait, balance, continence and neuropsychology, whereas gait is given twice, given in a formula possible to revice according to how many of the variables are accessable.

### Outcome assessment

As the SHQR did not until recently contain data about the 10 m walk test or neuropsychology test results, a modified (m) version of the iNPH scale introduced by Hellström et al. [7] was calculated. The miNPH scale has previously been introduced in a study of outcome based on data from the SHQR [16]. In accordance with the original iNPH scale, each ordinal scale score for gait, balance, and incontinence in the SHQR was converted into a continous domain score ranging from 0 (most severe state) to 100 (performance of age-matched healthy population) (Table 1) and transformed to an miNPH scale score according to Eq. (1) where gait performance is weighed twice, resulting in the same range for the miNPH scale score.

**Table 1:**
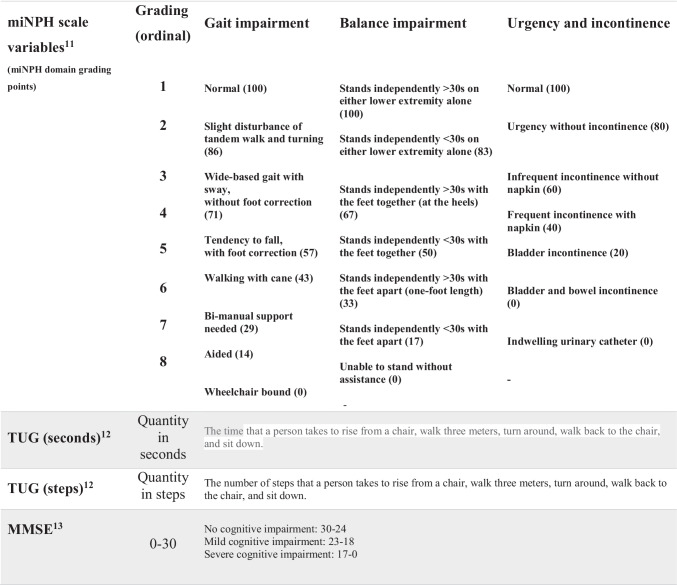
Rating scales and other clinical tests assessed and their associated gradings

**Abbreviations:** *miNPH scale*, modified idiopathic normal pressure hydrocephalus scale; *TUG*, Timed Up and Go test; *MMSE*, mini mental state examination

1 $$\frac{2\times \mathrm{Gait}\pm \mathrm{Balance}\pm \mathrm{Incontinence}}{4}$$

MiNPH scale scores were only calculated if all domains (gait, balance and incontinence) were reported in the registry. A significant improvement in the miNPH scale was defined as an increment of ≥ 5 points [7]. In addition, results on the Timed Up and Go (TUG) test [11] and the Mini Mental State Examination (MMSE) [5] were also used for analysis of outcome.

### Validation of the data in the SHQR

The SHQR was manually cross-checked for conformity with medical records and high patient coverage by audits between centres during the first years of start-up as well as during 2017–2018. Dedicated personnel at each centre are assigned the task of registering all hydrocephalus patients operated on in a structured way, ensuring high quality of included data. The concentration of all surgeries to a few centres also allows for high consistency in registrations and patient coverage rates.

### Statistics

Data are presented as means with standard deviation or medians with range and interquartile range (IQR) as appropriate. Differences between groups were analysed using the Wilcoxon rank sum test, Wilcoxon signed rank test, and the chi-squared test, as indicated. P-values are given with four decimals. Statistical analyses were performed with JMP (version 14.1.0, SAS Institute Inc, Cary, North Carolina, USA).

### Ethics approval

The study was approved by the Swedish Ethical Review Authority (Dnr 2019–02,542).

## Results

**Fig. 1:**
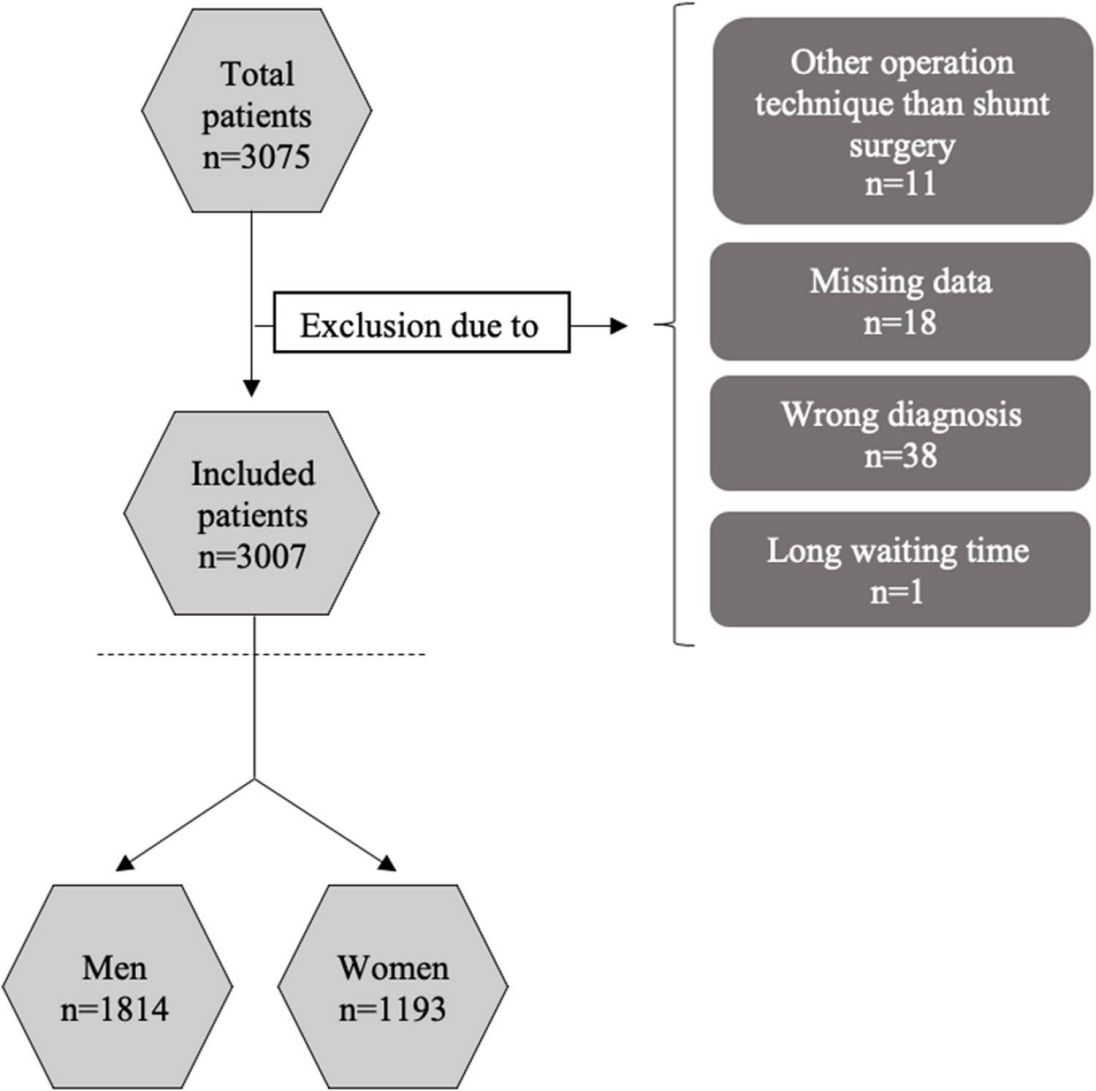
Flowchart showing inclusion and exclusion of patients in the study

Data on 3075 NPH patients were extracted from SHQR of which 3007 were accepted and included in the study. The reasons for exclusion are presented in Fig. 1. The analysis included 1814 (60.3%) men with a mean age of 74.3 (SD 6.6) years and 1193 (39.7%) women with a mean age of 74.2 (SD 7.1) years. Table 2 shows the distribution of age, sex and miNPH between the three waiting time groups. Number of patients having data on all three domains in the miNPH scale preoperatively as well as at 3 months of follow-up were 1279 (39.4% women), and at 12 months of follow-up 762 (41.9% women).

**Table 2:**
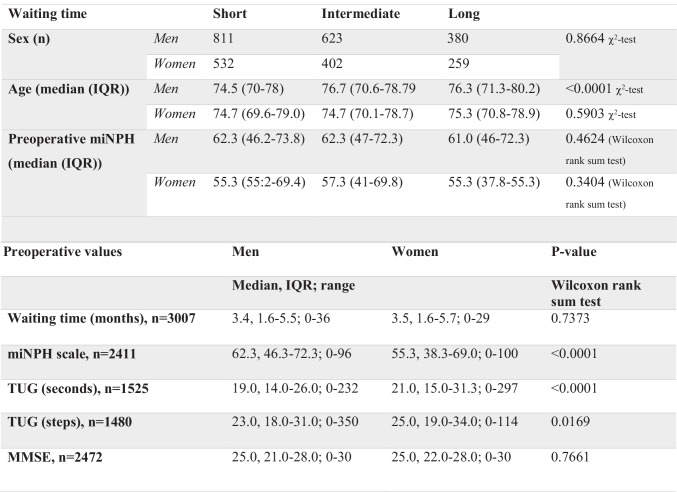
Patients included in the study

**Abbreviations:** *miNPH scale*, modified idiopathic normal pressure hydrocephalus scale; *TUG*, Timed Up and Go test; *MMSE*, mini mental state examination

The mean waiting time for surgery was 4.0 months (SD 3.5, median 3.4, IQR 1.6–5.6, range 0–36 months, n = 3007) with no difference between sexes (Table 2). In total, 45% had received surgery within 3 months from decision, and a fifth (21%) had a long waiting time (> 6 months). The median waiting time in the groups that received surgery within short, intermediate and long waiting time were 1.4 (IQR 0.5–2.2, n = 1343), 4.3 (IQR 3.6–5.1, n = 1025) and 7.7 (IQR 6.8–9.7, n = 639) months respectively. There was no difference between sexes for either of the groups (p-values 0.7966, 0.8408 and 0.8465 respectively, Wilcoxon rank sum test). Men performed slightly better preoperatively on the miNPH scale (p < 0.0001) and in the TUG test (p < 0.0001 and p = 0.0169), but not in MMSE (Table 2).

**Fig. 2:**
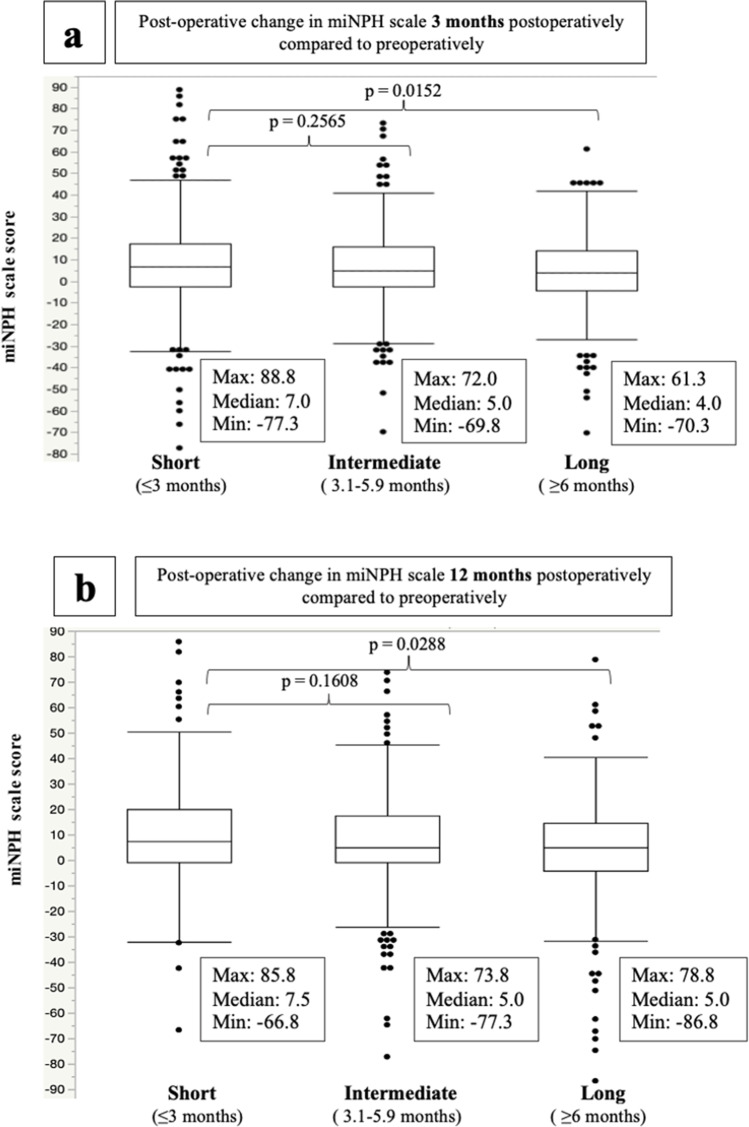
The postoperative change in miNPH scale scores after surgery in relation to waiting time. P-values given using Wilcoxon rank sum test

Figure 2 illustrates the change in miNPH scale at 6 and 12 months of follow-up, stratified for waiting time. Median improvement in those with short waiting time was more than 5 miNPH scale points at both 3 and 12 months of follow-up. Compared to this group, a lesser improvement was seen in those operated after a long waiting time at both 3 and 12 months of follow-up.

In Table 3 where pre- and postoperative findings for the whole cohort are presented, improvement in all parameters were found.

**Table 3:**
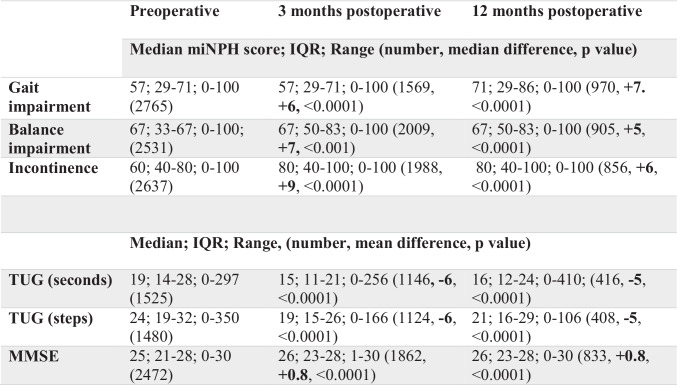
Postoperative outcome at 3 and 12 months for gait and balance impairment, incontinence, TUG (in both seconds and number of steps) and MMSE, paired comparisons with Wilcoxon Signed Rank Test

Table 4 illustrates the effect of waiting time on the outcome at 3 and 12 months of follow-up. All patients improved in regard to the miNPH scale at both follow-up points, though the improvement at both times was less pronounced among those who had a long waiting time as compared with those operated within 3 months (short waiting time) of decision. The same result was seen for TUG. In the same group of patients, there was no significant difference in outcome for those with waiting time in the interval of 3.1–5.9 months, compared with those with a short waiting time.

**Table 4:**
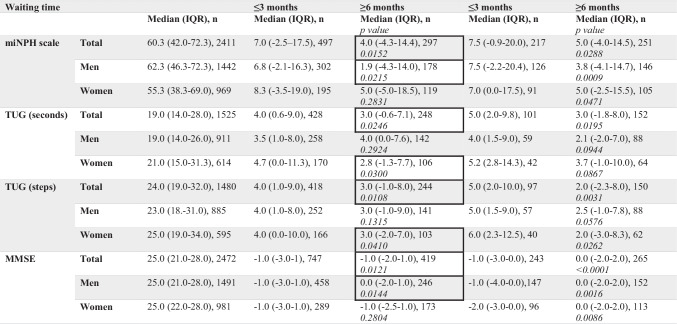
Postoperative change at 3 and 12 months of follow-up for the miNPH scale, TUG and MMSE in relation to preoperative values and waiting time. P-values relate to comparisons between outcome for patients with ≤ 3 and ≥ 6 months waiting time using the Wilcoxon Rank Sum Test. Highlighted are p-values < 0.05

Overall improvement defined as an increase in miNPH scale of ≥ 5 was found in 53% of the study population at 3 months of follow-up whereas an 55% improvement rate was seen at 12 months of follow-up.

**Fig. 3:**
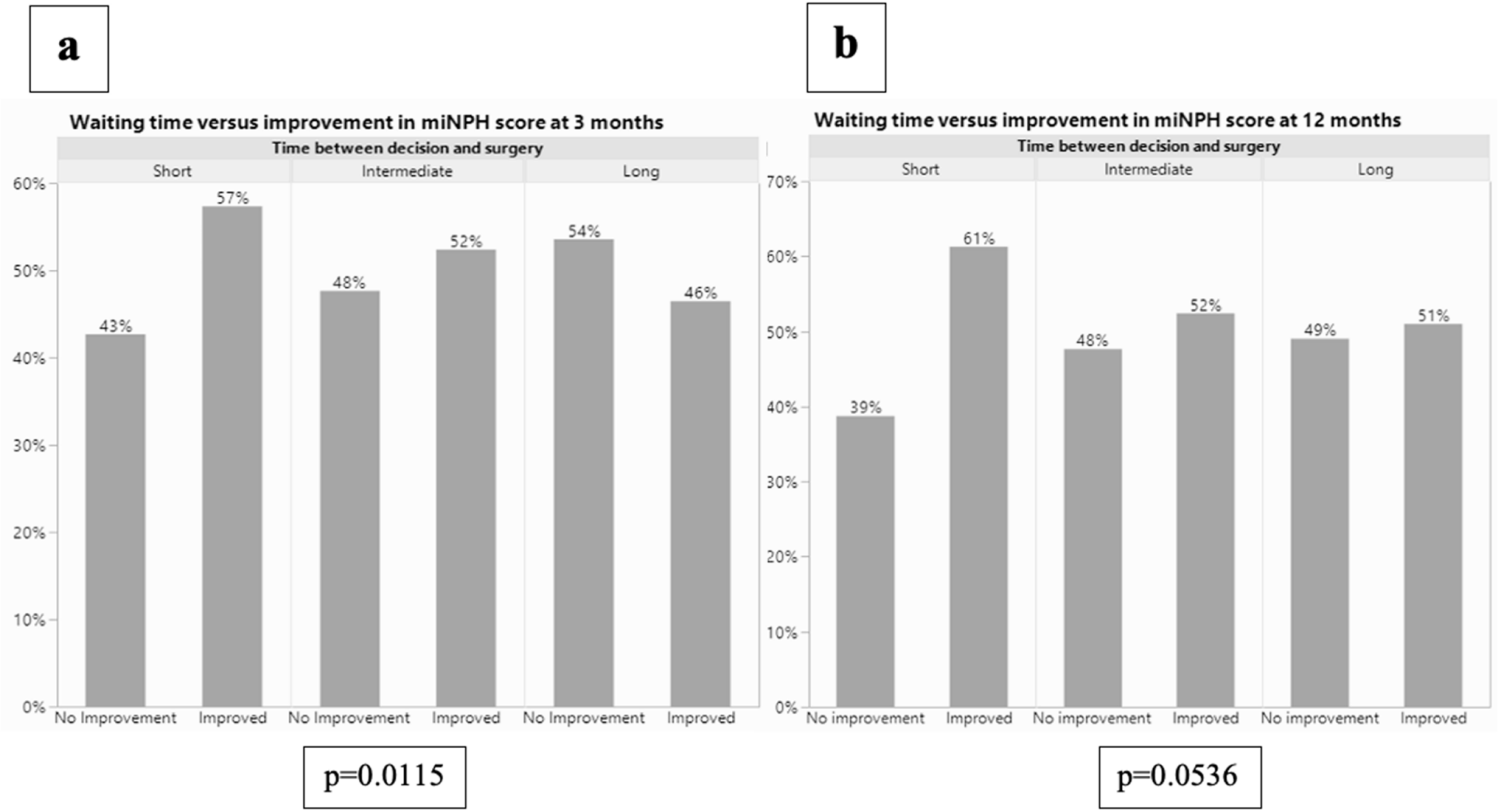
Percent improved and non-improved, defined as an increase in miNPH scale of ≥ 5. P-values are given using chi-squared test

Figure 3 illustrates the outcome defined as an improvement in the miNPH scale of ≥ 5 points, as defined by Hellström [7]. At 3 months of follow-up, a higher frequency of improvement is seen among those operated with a short waiting time (≤ 3 months) as compared with those operated with a long waiting time (≥ 6 months) (p = 0.0115). A similar result was seen at 12 months of follow-up (p = 0.0536).

## Discussion

This study shows that a longer waiting time for shunt surgery for patients with iNPH negatively influences the outcome. Patients who had a long waiting time (≥ 6 months) for surgery showed less pronounced improvement in all clinical measures at both 3 and 12 months of follow-up compared to patients with a short (≤ 3 months) waiting time. This finding was further reinforced by the finding of the same differences in the proportions of improved (≥ 5 miNPH points) patients, in relation to waiting time. Therefore, shunt surgery should probably be performed as soon as possible.

Our finding that time negatively influences the outcome if the waiting time is divided in ≤ 3 months versus ≥ 6 months was also seen in all other analysed outcome measures, which showed less improvement in the ≥ 6 month-group, at both 3- and 12-month follow-up. The same tendency was seen in the intermediate waiting time group; that earlier surgery gives a better outcome. There was no difference in sex, age or preoperative miNPH scale score between the waiting time groups, ensuring that the differences seen in clinical outcome was not due to these factors. Comparing data analysed as improved and non-improved defined as an increase in miNPH scale of ≥ 5, we saw the same result which further strengthens our conclusion.

Even if clinical improvement was seen regardless of when in time surgery was effectuated, our results support the notion that it is not ethically defendable to postpone the treatment, i.e. surgery, knowing that the outcome improves the earlier it is conducted.

To our knowledge, only one study, including few patients, by Andrén and co-workers has addressed the same question as ours [2]. Our population-based findings based on the registered patients operated for iNPH in Sweden confirm the findings by Andrén et al. They showed a decrease of the total score on the iNPH scale with ≥ 5 points for those with a prolonged waiting time to surgery, in 55% of the patients. Our study showed the same tendencies which further are seen at the follow-up post operatively at 3 and 12 months.

Our findings could well be expected since iNPH is a progressive disease, proposed causing irreversible brain damage if not treated promptly [2, 3, 18]. One should assume that a longer waiting time for surgery gives the disease more time for progression i.e. the longer the patient has to wait, the more advanced will the symptoms be at the time of surgery. Thus, impairing the prognosis. This same reasonable conclusion on waiting time and prognosis can be drawn for many other conditions e.g. bariatric surgery on obesitas, elective surgical lumbar discectomy and pain and surgery on oncological diseases [4, 12, 19].

Overall, irrespective of the waiting time, the patients seem to benefit from surgery confirming that shunt surgery is an effective treatment for iNPH as is known from many reports [7, 10, 13, 15].

When analysing the preoperative parameters, it is clear that women seem to come to diagnose with a more developed disease. The difference is most obvious when looking at the miNPH scale and not as great in TUG (seconds and steps) and MMSE. We also found that 20% more men than women had had shunt surgery. This is an interesting finding since most authors report that the frequency of iNPH is even with both sexes [1, 20]. Women do though improve from shunt surgery as much as men and show the same negative influence of waiting time [10]. To elucidate the reasons for these differences between the sexes, a future study must be performed. It is compelling to discuss whether women generally seek medical attention at a later stage of disease than men do. If that is the case, more equal healthcare and shortening the access to care, might increase improvement for iNPH.

Interestingly, though a smaller improvement in outcome can be seen if the intervention is offered later than 3 months after taking the decision to operate, the outcome at 12-month follow-up shows a further improvement in most parameters analysed, than at the 3-month follow-up. Thus, the ultimate outcome is achieved if intervention is offered within 3 months. We noted that the improvement is not only sustained over time but has a tendency to increase at least during the first post-operative year.

The overall proportion of improved patients irrespectively of waiting time reported here was 53% and 55% at 3 and 12 months respectively, which is lower than what is reported in many single-centre studies including the European multicentre study [10] but similar to what is earlier reported from the SHQR [16]. This could be due to less sensitivity of the ordinal scales included in the miNPH scale but also to a more unselected and heterogeneous study sample.

In Sweden, there is a national agreement on a so called “health care guaranty” which means that the patients should be guaranteed to receive the accurate type of health care within 3 months. For iNPH patients, this study shows the importance of fulfilling this time limit and performing shunt surgery within 3 months, to obtain an optimal clinical result.

A strength of this study is that the SHQR is population based and has been sustained over time, enabling important analyses on a large, unselected patient material. One limitation, though, is that the registry does not include all iNPH patients operated on in Sweden, i.e. about 20% are missing. Most likely, this did not affect the outcome of the study since in periods, some surgical centres have not been reporting any data, i.e. all patient data from a specific centre is missing over a certain time. The reporting centres have thus either reported all their patients operated or none, and so no selection bias has been introduced in the reported patients. From this, it follows that patients in the SHQR are likely to constitute a good representation of the Swedish iNPH population. In SHQR, the TUG test was first registered in 2010; as a result, the number of patients evaluated with this test was lower than those assessed with MMSE or the ordinal test that are part of the miNPH scale. Likewise, the number of patients that could be assessed 12 months after surgery was fewer than at 3-month follow-up due to lack of routine clinical 12-month follow-up at some centres.

## Conclusion

Our population-based study on patients with iNPH shows that shunt surgery should be performed within 3 months of decision to surgery, to attain the best clinical outcome. It also shows that with increasing waiting time, the outcome is less favourable. It is therefore of importance to keep the waiting time as short as possible in order to optimise treatment effects.

## Data Availability

The original material can be requested from the SHQR.

## Code availability

N/A.
